# 1-(3,5-Dimethyl­phen­yl)-4,5-dimethyl-2-phenyl-1*H*-imidazole hemihydrate

**DOI:** 10.1107/S1600536810039784

**Published:** 2010-10-09

**Authors:** P. Gayathri, A. Thiruvalluvar, N. Srinivasan, J. Jayabharathi, R. J. Butcher

**Affiliations:** a PG Research Department of Physics, Rajah Serfoji Government College (Autonomous), Thanjavur 613 005, Tamilnadu, India; b Department of Chemistry, Annamalai University, Annamalai Nagar 608 002, Tamilnadu, India; c Department of Chemistry, Howard University, 525 College Street NW, Washington, DC 20059, USA

## Abstract

In the title compound, C_19_H_20_N_2_·0.5H_2_O, the imidazole ring is essentially planar [maximum deviation = 0.005 (1) Å]. The imidazole ring makes dihedral angles of 67.46 (10) and 23.10 (11)° with the attached benzene and phenyl rings, respectively. The dihedral angle between the benzene and phenyl rings is 68.22 (10)°. Inter­molecular O—H⋯N and C—H⋯N hydrogen bonds are found in the crystal structure.

## Related literature

For pharmacological properties of imidazole compounds, see: Lombardino & Wiseman (1974 ▶); For the applications of substituted imidazoles, see: Maier *et al.* (1989*a* ▶,*b* ▶). For the synthesis of imidazoles, see: Welton (1999 ▶); Hermann & Kocher (1997 ▶). For imidazole derivatives as anti­cancer agents, see: Krezel (1998 ▶). For related structures and applications of imidazole derivatives, see: Gayathri *et al.* (2010*a* ▶,*b* ▶,*c* ▶,*d* ▶).
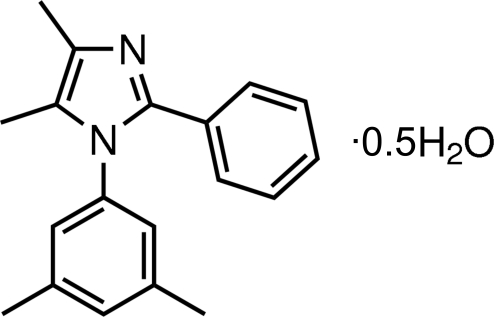


## Experimental

### 

#### Crystal data

C_19_H_20_N_2_·0.5H_2_O
*M* *_r_* = 285.38
Orthorhombic, 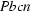

*a* = 16.7611 (2) Å
*b* = 11.5467 (2) Å
*c* = 16.6563 (2) Å
*V* = 3223.58 (8) Å^3^
*Z* = 8
Cu *K*α radiation
μ = 0.55 mm^−1^
*T* = 123 K
0.47 × 0.38 × 0.18 mm

#### Data collection

Oxford Diffraction Xcalibur Ruby Gemini diffractometer
Absorption correction: multi-scan (*CrysAlis PRO*; Oxford Diffraction, 2010 ▶) *T*_min_ = 0.818, *T*_max_ = 1.000
7791 measured reflections
3193 independent reflections
2630 reflections with *I* > 2σ(*I*)
*R*_int_ = 0.020

#### Refinement

*R*[*F* ^2^ > 2σ(*F* ^2^)] = 0.062
*wR*(*F* ^2^) = 0.171
*S* = 1.08
3193 reflections
203 parameters
H atoms treated by a mixture of independent and constrained refinement
Δρ_max_ = 0.56 e Å^−3^
Δρ_min_ = −0.33 e Å^−3^

### 

Data collection: *CrysAlis PRO* (Oxford Diffraction, 2010 ▶); cell refinement: *CrysAlis PRO*; data reduction: *CrysAlis PRO*; program(s) used to solve structure: *SHELXS97* (Sheldrick, 2008 ▶); program(s) used to refine structure: *SHELXL97* (Sheldrick, 2008 ▶); molecular graphics: *ORTEP-3* (Farrugia, 1997 ▶); software used to prepare material for publication: *PLATON* (Spek, 2009 ▶).

## Supplementary Material

Crystal structure: contains datablocks global, I. DOI: 10.1107/S1600536810039784/hg2722sup1.cif

Structure factors: contains datablocks I. DOI: 10.1107/S1600536810039784/hg2722Isup2.hkl

Additional supplementary materials:  crystallographic information; 3D view; checkCIF report

## Figures and Tables

**Table 1:** Hydrogen-bond geometry (Å, °)

*D*—H⋯*A*	*D*—H	H⋯*A*	*D*⋯*A*	*D*—H⋯*A*
O1*W*—H1*W*⋯N3^i^	0.88 (5)	2.11 (5)	2.937 (2)	155 (4)
C12—H12⋯N3^ii^	0.95	2.58	3.388 (3)	144

Symmetry codes: (i) 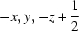; (ii) 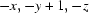.

**Fig. 1.**
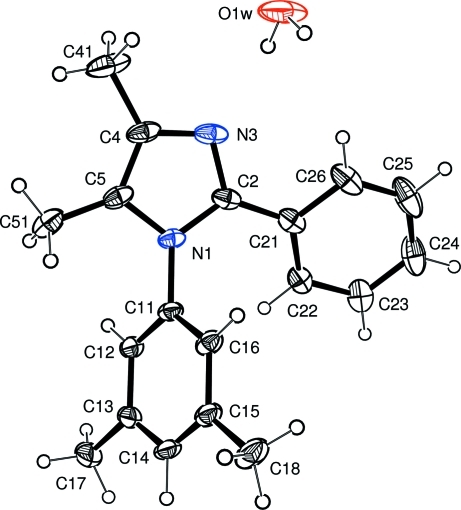
The molecular structure of the title compound, showing the atom-numbering scheme and displacement ellipsoids drawn at the 30% probability level. H atoms are shown as small spheres of arbitrary radius.

**Fig. 2.**
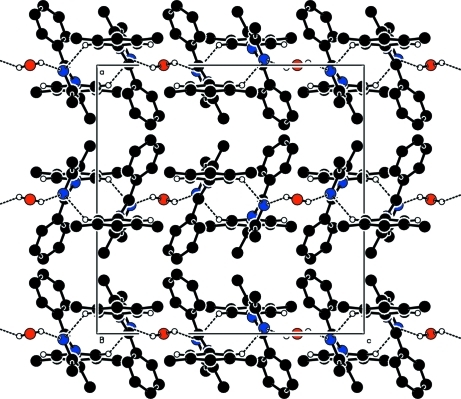
The packing of the title compound, viewed down the b axis. Dashed lines indicate hydrogen bonds. H atoms not involved in hydrogen bonding have been omitted.

## supplementary crystallographic information

### Comment

Compounds with an imidazole ring system have many pharmacological properties and play important roles in biochemical processes (Lombardino & Wiseman (1974)). Many of the substituted imidazoles are known as inhibitors of fungicides and herbicides, plant growth regulators and therapeutic agents (Maier *et al.* (1989*a*,*b*). Recent advances in green chemistry and organometallic chemistry have extended the boundary of imidazoles to the synthesis and application of a large class of imidazoles as ionic liquids and imidazole related N-heterocyclic carbenes (Welton (1999) and Hermann & Kocher (1997)). Imidazole derivatives are also used as as potential anticancer agents (Krezel (1998)). As part of our research (Gayathri *et al.*, (2010*a*,*b*,*c*,*d*)), we have synthesized the title compound (I) and report its crystal structure here.

In the title compound (Fig. 1), C_19_H_20_N_2_.H_2_O, the imidazole ring is essentially planar [maximum deviation of 0.005 (1) Å for N3]. The imidazole ring makes dihedral angles of 67.46 (10) and 23.10 (11)° with the benzene ring (C11—C16) attached to N1 and phenyl ring (C21—C26) attached to C2 respectively. The dihedral angle between the benzene and phenyl rings is 68.22 (10)°. Intermolecular O1W—H1W···N3 and C12—H12···N3 hydrogen bonds are found in the crystal structure (Table 1, Fig. 2).

### Experimental

To pure butane-2,3-dione (1.48 g, 15 mmol) in ethanol (10 ml), 3,5-xylidine (1.8 g, 15 mmol), ammonium acetate (1.15 g, 15 mmol) and benzaldehyde (1.5 g, 15 mmol) was added about 1 h by maintaining the temperature at 333 K. The reaction mixture was refluxed for 7 days and extracted with dichloromethane. The solid separated was purified by column chromatography using hexane: ethyl acetate as the eluent. Yield: 1.91 g (46%).

### Refinement

H1W attached to O1W was located in a difference Fourier map and refined freely. Another H atom attached to O1W falls on a symmetry (-*x*, *y*, 1/2 - *z*). Remaining H atoms were positioned geometrically and allowed to ride on their parent atoms, with C—H = 0.95 - 0.98 Å; *U*_iso_(H) = k*U*_eq_(C), where k = 1.5 for methyl and 1.2 for all other H atoms.

### Crystal data

**Table:**

C_19_H_20_N_2_·0.5H_2_O	*D*_x_ = 1.176 Mg m^−^^3^
*M**_r_* = 285.38	Melting point: 415 K
Orthorhombic, *P**b**c**n*	Cu *K*α radiation, λ = 1.54184 Å
Hall symbol: -P 2n 2ab	Cell parameters from 5434 reflections
*a* = 16.7611 (2) Å	θ = 5.2–28.5°
*b* = 11.5467 (2) Å	µ = 0.55 mm^−^^1^
*c* = 16.6563 (2) Å	*T* = 123 K
*V* = 3223.58 (8) Å^3^	Prism, colourless
*Z* = 8	0.47 × 0.38 × 0.18 mm
*F*(000) = 1224	

### Data collection

**Table:**

Oxford Diffraction Xcalibur Ruby Gemini diffractometer	3193 independent reflections
Radiation source: Enhance (Cu) X-ray Source	2630 reflections with *I* > 2σ(*I*)
graphite	*R*_int_ = 0.020
Detector resolution: 10.5081 pixels mm^-1^	θ_max_ = 74.2°, θ_min_ = 5.3°
ω scans	*h* = −20→20
Absorption correction: multi-scan (*CrysAlis PRO*; Oxford Diffraction, 2010)	*k* = −13→8
*T*_min_ = 0.818, *T*_max_ = 1.000	*l* = −20→18
7791 measured reflections	

### Refinement

**Table:**

Refinement on *F*^2^	Primary atom site location: structure-invariant direct methods
Least-squares matrix: full	Secondary atom site location: difference Fourier map
*R*[*F*^2^ > 2σ(*F*^2^)] = 0.062	Hydrogen site location: inferred from neighbouring sites
*wR*(*F*^2^) = 0.171	H atoms treated by a mixture of independent and constrained refinement
*S* = 1.08	*w* = 1/[σ^2^(*F*_o_^2^) + (0.0758*P*)^2^ + 2.1555*P*] where *P* = (*F*_o_^2^ + 2*F*_c_^2^)/3
3193 reflections	(Δ/σ)_max_ = 0.001
203 parameters	Δρ_max_ = 0.56 e Å^−^^3^
0 restraints	Δρ_min_ = −0.33 e Å^−^^3^

### Special details

**Table:**

Geometry. Bond distances, angles *etc*. have been calculated using the rounded fractional coordinates. All su's are estimated from the variances of the (full) variance-covariance matrix. The cell e.s.d.'s are taken into account in the estimation of distances, angles and torsion angles
Refinement. Refinement of *F*^2^ against ALL reflections. The weighted *R*-factor *wR* and goodness of fit *S* are based on *F*^2^, conventional *R*-factors *R* are based on *F*, with *F* set to zero for negative *F*^2^. The threshold expression of *F*^2^ > 2σ(*F*^2^) is used only for calculating *R*-factors(gt) *etc*. and is not relevant to the choice of reflections for refinement. *R*-factors based on *F*^2^ are statistically about twice as large as those based on *F*, and *R*- factors based on ALL data will be even larger.

### Fractional atomic coordinates and isotropic or equivalent isotropic displacement parameters (Å^2^)

**Table:**

	*x*	*y*	*z*	*U*_iso_*/*U*_eq_	
N1	0.06312 (10)	0.58209 (13)	0.08298 (9)	0.0337 (5)	
N3	0.02135 (14)	0.41115 (14)	0.12586 (9)	0.0486 (6)	
C2	0.00259 (13)	0.52288 (16)	0.11985 (10)	0.0373 (6)	
C4	0.09630 (17)	0.39935 (18)	0.09289 (12)	0.0478 (7)	
C5	0.12333 (13)	0.50362 (17)	0.06558 (11)	0.0405 (6)	
C11	0.07375 (11)	0.70592 (16)	0.07891 (12)	0.0320 (5)	
C12	0.07941 (11)	0.75929 (17)	0.00507 (13)	0.0366 (6)	
C13	0.09222 (12)	0.87905 (18)	0.00254 (15)	0.0457 (7)	
C14	0.09830 (12)	0.93880 (18)	0.07557 (17)	0.0513 (8)	
C15	0.09275 (11)	0.88545 (18)	0.14936 (15)	0.0437 (7)	
C16	0.08061 (11)	0.76691 (17)	0.15073 (13)	0.0378 (6)	
C17	0.09981 (16)	0.9395 (2)	−0.07700 (17)	0.0618 (9)	
C18	0.10108 (15)	0.9525 (2)	0.22687 (18)	0.0622 (9)	
C21	−0.07307 (12)	0.57105 (19)	0.14706 (11)	0.0394 (6)	
C22	−0.10598 (12)	0.67293 (19)	0.11567 (12)	0.0410 (6)	
C23	−0.17918 (13)	0.7120 (3)	0.14236 (13)	0.0529 (8)	
C24	−0.22085 (14)	0.6511 (3)	0.20103 (14)	0.0654 (11)	
C25	−0.18898 (15)	0.5512 (3)	0.23230 (14)	0.0682 (12)	
C26	−0.11644 (14)	0.5112 (2)	0.20583 (13)	0.0540 (8)	
C41	0.1384 (2)	0.2843 (2)	0.09236 (15)	0.0750 (12)	
C51	0.19881 (14)	0.5369 (2)	0.02610 (16)	0.0526 (8)	
O1W	0.00000	0.2332 (2)	0.25000	0.126 (2)	
H12	0.07471	0.71571	−0.04308	0.0439*	
H14	0.10666	1.02015	0.07411	0.0616*	
H16	0.07696	0.72722	0.20056	0.0453*	
H17A	0.06867	0.89766	−0.11750	0.0927*	
H17B	0.07968	1.01885	−0.07221	0.0927*	
H17C	0.15601	0.94126	−0.09315	0.0927*	
H18A	0.08520	1.03316	0.21803	0.0933*	
H18B	0.06672	0.91783	0.26797	0.0933*	
H18C	0.15672	0.94989	0.24485	0.0933*	
H22	−0.07782	0.71534	0.07585	0.0492*	
H23	−0.20127	0.78087	0.12053	0.0635*	
H24	−0.27110	0.67841	0.21935	0.0785*	
H25	−0.21725	0.50966	0.27246	0.0818*	
H26	−0.09530	0.44170	0.22774	0.0648*	
H41A	0.19154	0.29351	0.06879	0.1122*	
H41B	0.14340	0.25567	0.14751	0.1122*	
H41C	0.10750	0.22885	0.06044	0.1122*	
H51A	0.18815	0.55867	−0.02975	0.0789*	
H51B	0.22245	0.60282	0.05453	0.0789*	
H51C	0.23592	0.47135	0.02731	0.0789*	
H1W	0.009 (3)	0.280 (4)	0.291 (3)	0.138 (17)*	

### Atomic displacement parameters (Å^2^)

**Table:**

	*U*^11^	*U*^22^	*U*^33^	*U*^12^	*U*^13^	*U*^23^
N1	0.0457 (9)	0.0230 (8)	0.0325 (8)	−0.0005 (6)	−0.0068 (7)	−0.0043 (6)
N3	0.0938 (15)	0.0256 (8)	0.0263 (8)	−0.0062 (9)	−0.0095 (9)	−0.0004 (6)
C2	0.0581 (12)	0.0287 (10)	0.0251 (8)	−0.0106 (9)	−0.0087 (8)	−0.0003 (7)
C4	0.0891 (18)	0.0264 (10)	0.0280 (9)	0.0084 (10)	−0.0105 (10)	−0.0056 (7)
C5	0.0575 (12)	0.0305 (10)	0.0334 (10)	0.0089 (9)	−0.0118 (9)	−0.0095 (8)
C11	0.0301 (9)	0.0211 (9)	0.0447 (10)	−0.0013 (7)	−0.0033 (8)	−0.0052 (7)
C12	0.0339 (9)	0.0286 (10)	0.0472 (11)	0.0000 (7)	0.0032 (8)	−0.0027 (8)
C13	0.0348 (10)	0.0308 (10)	0.0716 (15)	0.0026 (8)	0.0136 (10)	0.0091 (10)
C14	0.0373 (11)	0.0228 (10)	0.0939 (19)	−0.0027 (8)	0.0135 (11)	−0.0146 (11)
C15	0.0325 (10)	0.0304 (10)	0.0682 (14)	−0.0029 (8)	0.0044 (9)	−0.0163 (10)
C16	0.0318 (9)	0.0329 (10)	0.0486 (11)	0.0011 (8)	−0.0046 (8)	−0.0120 (8)
C17	0.0595 (15)	0.0423 (13)	0.0835 (19)	0.0066 (11)	0.0225 (13)	0.0217 (12)
C18	0.0514 (13)	0.0480 (14)	0.0872 (19)	−0.0053 (11)	0.0009 (13)	−0.0374 (13)
C21	0.0488 (11)	0.0423 (11)	0.0270 (9)	−0.0163 (9)	−0.0087 (8)	0.0002 (8)
C22	0.0420 (10)	0.0500 (12)	0.0311 (9)	−0.0106 (9)	−0.0029 (8)	−0.0017 (9)
C23	0.0415 (11)	0.0765 (17)	0.0407 (11)	−0.0037 (11)	−0.0059 (9)	−0.0032 (11)
C24	0.0373 (11)	0.118 (3)	0.0409 (12)	−0.0158 (14)	0.0000 (10)	−0.0047 (14)
C25	0.0509 (14)	0.117 (3)	0.0366 (12)	−0.0310 (15)	−0.0069 (10)	0.0178 (14)
C26	0.0594 (14)	0.0665 (16)	0.0361 (11)	−0.0281 (12)	−0.0119 (10)	0.0113 (10)
C41	0.147 (3)	0.0334 (12)	0.0445 (13)	0.0293 (16)	−0.0177 (16)	−0.0015 (10)
C51	0.0514 (13)	0.0427 (12)	0.0637 (14)	0.0120 (10)	−0.0073 (11)	−0.0182 (11)
O1W	0.300 (7)	0.0283 (13)	0.0503 (16)	0.0000	0.041 (3)	0.0000

### Geometric parameters (Å, °)

**Table:**

O1W—H1W^i^	0.88 (5)	C24—C25	1.374 (5)
O1W—H1W	0.88 (5)	C25—C26	1.373 (4)
N1—C5	1.387 (3)	C12—H12	0.9500
N1—C2	1.369 (3)	C14—H14	0.9500
N1—C11	1.443 (2)	C16—H16	0.9500
N3—C4	1.378 (4)	C17—H17B	0.9800
N3—C2	1.332 (3)	C17—H17A	0.9800
C2—C21	1.457 (3)	C17—H17C	0.9800
C4—C41	1.504 (3)	C18—H18C	0.9800
C4—C5	1.365 (3)	C18—H18B	0.9800
C5—C51	1.477 (3)	C18—H18A	0.9800
C11—C12	1.379 (3)	C22—H22	0.9500
C11—C16	1.393 (3)	C23—H23	0.9500
C12—C13	1.400 (3)	C24—H24	0.9500
C13—C17	1.503 (4)	C25—H25	0.9500
C13—C14	1.402 (4)	C26—H26	0.9500
C14—C15	1.378 (4)	C41—H41B	0.9800
C15—C18	1.512 (4)	C41—H41C	0.9800
C15—C16	1.384 (3)	C41—H41A	0.9800
C21—C22	1.401 (3)	C51—H51C	0.9800
C21—C26	1.402 (3)	C51—H51A	0.9800
C22—C23	1.381 (3)	C51—H51B	0.9800
C23—C24	1.392 (4)		
			
O1W···N3^i^	2.937 (2)	C26···H16^i^	3.0200
O1W···N3	2.937 (2)	C41···H22^ii^	2.9800
O1W···H26	2.9100	C41···H51C	2.9200
O1W···H26^i^	2.9100	C51···H41A	2.9000
O1W···H17A^ii^	2.9100	H1W···N3^i^	2.11 (5)
O1W···H18A^iii^	2.7700	H1W···C4^i^	2.96 (5)
O1W···H18A^iv^	2.7700	H1W···H26^i^	2.3800
O1W···H17A^v^	2.9100	H12···H17A	2.4400
N3···O1W	2.937 (2)	H12···N3^ii^	2.5800
N3···O1W	2.937 (2)	H14···H41C^xii^	2.4200
N3···C26^i^	3.426 (3)	H14···H17B	2.4800
N3···C12^ii^	3.388 (3)	H14···H18A	2.4300
N1···H22	2.8200	H16···C2	2.9900
N3···H26	2.6100	H16···H18B	2.4800
N3···H1W^i^	2.11 (5)	H16···C24^i^	3.0500
N3···H26^i^	2.7600	H16···C25^i^	2.9800
N3···H12^ii^	2.5800	H16···C26^i^	3.0200
C2···C26^i^	3.477 (3)	H17A···O1W^xiii^	2.9100
C4···C22^ii^	3.576 (3)	H17A···H12	2.4400
C5···C25^i^	3.584 (3)	H17A···O1W^ii^	2.9100
C11···C22	3.098 (3)	H17B···C14^ix^	3.0200
C12···N3^ii^	3.388 (3)	H17B···H14	2.4800
C12···C51	3.274 (3)	H17C···C24^xiv^	2.9400
C16···C22	3.362 (3)	H18A···O1W^xii^	2.7700
C16···C21	3.428 (3)	H18A···O1W^xv^	2.7700
C17···C18^vi^	3.497 (4)	H18A···H14	2.4300
C18···C18^i^	3.475 (4)	H18B···C15^i^	3.0300
C18···C17^vii^	3.497 (4)	H18B···H16	2.4800
C21···C16	3.428 (3)	H18B···C18^i^	2.8400
C22···C41^ii^	3.542 (3)	H18B···H18B^i^	2.3200
C22···C4^ii^	3.576 (3)	H18C···C25^xvi^	2.8600
C22···C16	3.362 (3)	H18C···H25^xvi^	2.2400
C22···C11	3.098 (3)	H22···C16	2.9900
C25···C5^i^	3.584 (3)	H22···C41^ii^	2.9800
C26···N3^i^	3.426 (3)	H22···N1	2.8200
C26···C2^i^	3.477 (3)	H22···C11	2.5400
C41···C22^ii^	3.542 (3)	H22···H41C^ii^	2.4100
C51···C12	3.274 (3)	H22···C12	2.9300
C2···H16	2.9900	H25···H18C^xi^	2.2400
C4···H1W^i^	2.96 (5)	H26···O1W	2.9100
C4···H26^i^	3.0300	H26···C4^i^	3.0300
C11···H51B	2.7900	H26···H1W^i^	2.3800
C11···H22	2.5400	H26···O1W	2.9100
C12···H51A	3.0000	H26···N3^i^	2.7600
C12···H22	2.9300	H26···N3	2.6100
C13···H51C^viii^	3.1000	H41A···C51	2.9000
C14···H51C^viii^	2.9200	H41A···H51C	2.2900
C14···H17B^ix^	3.0200	H41C···H14^iv^	2.4200
C15···H18B^i^	3.0300	H41C···H22^ii^	2.4100
C16···H22	2.9900	H51A···C12	3.0000
C18···H18B^i^	2.8400	H51B···C11	2.7900
C24···H17C^x^	2.9400	H51C···H41A	2.2900
C24···H16^i^	3.0500	H51C···C13^xvii^	3.1000
C25···H18C^xi^	2.8600	H51C···C14^xvii^	2.9200
C25···H16^i^	2.9800	H51C···C41	2.9200
			
H1W—O1W—H1W^i^	105 (4)	C13—C14—H14	118.00
C2—N1—C11	127.42 (16)	C11—C16—H16	120.00
C5—N1—C11	123.22 (16)	C15—C16—H16	120.00
C2—N1—C5	107.86 (15)	C13—C17—H17B	109.00
C2—N3—C4	106.33 (18)	C13—C17—H17C	109.00
N1—C2—C21	126.44 (17)	C13—C17—H17A	109.00
N3—C2—C21	123.50 (19)	H17A—C17—H17C	109.00
N1—C2—N3	110.03 (18)	H17B—C17—H17C	110.00
N3—C4—C41	121.2 (2)	H17A—C17—H17B	109.00
C5—C4—C41	128.4 (2)	C15—C18—H18B	109.00
N3—C4—C5	110.39 (19)	C15—C18—H18C	109.00
N1—C5—C51	123.13 (18)	C15—C18—H18A	109.00
C4—C5—C51	131.5 (2)	H18A—C18—H18C	109.00
N1—C5—C4	105.38 (19)	H18B—C18—H18C	109.00
N1—C11—C16	118.10 (17)	H18A—C18—H18B	109.00
C12—C11—C16	122.30 (18)	C23—C22—H22	120.00
N1—C11—C12	119.56 (17)	C21—C22—H22	120.00
C11—C12—C13	118.6 (2)	C22—C23—H23	120.00
C12—C13—C17	119.9 (2)	C24—C23—H23	120.00
C14—C13—C17	122.0 (2)	C25—C24—H24	120.00
C12—C13—C14	118.1 (2)	C23—C24—H24	120.00
C13—C14—C15	123.3 (2)	C24—C25—H25	120.00
C14—C15—C18	121.8 (2)	C26—C25—H25	120.00
C16—C15—C18	120.4 (2)	C25—C26—H26	119.00
C14—C15—C16	117.8 (2)	C21—C26—H26	119.00
C11—C16—C15	119.9 (2)	C4—C41—H41A	109.00
C2—C21—C22	123.18 (18)	C4—C41—H41B	109.00
C2—C21—C26	118.72 (19)	H41A—C41—H41B	110.00
C22—C21—C26	118.08 (19)	H41A—C41—H41C	110.00
C21—C22—C23	120.3 (2)	C4—C41—H41C	109.00
C22—C23—C24	120.5 (3)	H41B—C41—H41C	109.00
C23—C24—C25	119.7 (2)	C5—C51—H51B	109.00
C24—C25—C26	120.3 (2)	C5—C51—H51C	109.00
C21—C26—C25	121.2 (2)	C5—C51—H51A	109.00
C11—C12—H12	121.00	H51A—C51—H51C	109.00
C13—C12—H12	121.00	H51B—C51—H51C	109.00
C15—C14—H14	118.00	H51A—C51—H51B	109.00
			
C5—N1—C2—N3	−0.6 (2)	C41—C4—C5—N1	−177.7 (2)
C5—N1—C2—C21	−178.74 (17)	C41—C4—C5—C51	1.8 (4)
C11—N1—C2—N3	−166.83 (17)	N1—C11—C12—C13	−178.02 (17)
C11—N1—C2—C21	15.0 (3)	C16—C11—C12—C13	−0.2 (3)
C2—N1—C5—C4	0.1 (2)	N1—C11—C16—C15	178.49 (17)
C2—N1—C5—C51	−179.45 (19)	C12—C11—C16—C15	0.7 (3)
C11—N1—C5—C4	167.03 (17)	C11—C12—C13—C14	−0.3 (3)
C11—N1—C5—C51	−12.5 (3)	C11—C12—C13—C17	179.14 (19)
C2—N1—C11—C12	−122.8 (2)	C12—C13—C14—C15	0.4 (3)
C2—N1—C11—C16	59.3 (3)	C17—C13—C14—C15	−179.0 (2)
C5—N1—C11—C12	72.8 (2)	C13—C14—C15—C16	0.0 (3)
C5—N1—C11—C16	−105.1 (2)	C13—C14—C15—C18	178.9 (2)
C4—N3—C2—N1	0.8 (2)	C14—C15—C16—C11	−0.6 (3)
C4—N3—C2—C21	179.07 (18)	C18—C15—C16—C11	−179.42 (18)
C2—N3—C4—C5	−0.8 (2)	C2—C21—C22—C23	178.0 (2)
C2—N3—C4—C41	177.5 (2)	C26—C21—C22—C23	−0.1 (3)
N1—C2—C21—C22	22.9 (3)	C2—C21—C26—C25	−178.5 (2)
N1—C2—C21—C26	−158.99 (19)	C22—C21—C26—C25	−0.3 (3)
N3—C2—C21—C22	−155.0 (2)	C21—C22—C23—C24	0.4 (4)
N3—C2—C21—C26	23.1 (3)	C22—C23—C24—C25	−0.3 (4)
N3—C4—C5—N1	0.5 (2)	C23—C24—C25—C26	−0.1 (4)
N3—C4—C5—C51	179.9 (2)	C24—C25—C26—C21	0.4 (4)

Symmetry codes: (i) −*x*, *y*, −*z*+1/2; (ii) −*x*, −*y*+1, −*z*; (iii) −*x*, *y*−1, −*z*+1/2; (iv) *x*, *y*−1, *z*; (v) *x*, −*y*+1, *z*+1/2; (vi) *x*, −*y*+2, *z*−1/2; (vii) *x*, −*y*+2, *z*+1/2; (viii) −*x*+1/2, *y*+1/2, *z*; (ix) −*x*, −*y*+2, −*z*; (x) *x*−1/2, −*y*+3/2, −*z*; (xi) *x*−1/2, *y*−1/2, −*z*+1/2; (xii) *x*, *y*+1, *z*; (xiii) *x*, −*y*+1, *z*−1/2; (xiv) *x*+1/2, −*y*+3/2, −*z*; (xv) −*x*, *y*+1, −*z*+1/2; (xvi) *x*+1/2, *y*+1/2, −*z*+1/2; (xvii) −*x*+1/2, *y*−1/2, *z*.

### Hydrogen-bond geometry (Å, °)

**Table:**

*D*—H···*A*	*D*—H	H···*A*	*D*···*A*	*D*—H···*A*
O1W—H1W···N3^i^	0.88 (5)	2.11 (5)	2.937 (2)	155 (4)
C12—H12···N3^ii^	0.95	2.58	3.388 (3)	144

Symmetry codes: (i) −*x*, *y*, −*z*+1/2; (ii) −*x*, −*y*+1, −*z*.
